# Snakes and ladders: A qualitative study understanding the active ingredients of social interaction around the use of audit and feedback

**DOI:** 10.1093/tbm/ibac114

**Published:** 2023-01-24

**Authors:** Laura Desveaux, Marlena Dang Nguyen, Noah Michael Ivers, Kimberly Devotta, Tara Upshaw, Noor Ramji, Karen Weyman, Tara Kiran

**Affiliations:** Institute for Better Health, Trillium Health Partners, 100 Queensway West, Mississauga, Ontario, Canada; Women’s College Hospital Institute for Health Systems Solutions and Virtual Care, 76 Grenville Ave Toronto, Toronto, Ontario, Canada; Institute for Health Policy, Management & Evaluation, University of Toronto, 155 College St, Toronto, Ontario, Canada; Women’s College Hospital Institute for Health Systems Solutions and Virtual Care, 76 Grenville Ave Toronto, Toronto, Ontario, Canada; Women’s College Hospital Institute for Health Systems Solutions and Virtual Care, 76 Grenville Ave Toronto, Toronto, Ontario, Canada; Department of Family and Community Medicine, University of Toronto, 500 University Avenue, Toronto, Canada; MAP Centre for Urban Health Solutions, Li Ka Shing Knowledge Institute of St. Michael’s Hospital, 30 Bond Street, Toronto, Canada; Dalla Lana School of Public Health, University of Toronto, 155 College St, Toronto, Canada; MAP Centre for Urban Health Solutions, Li Ka Shing Knowledge Institute of St. Michael’s Hospital, 30 Bond Street, Toronto, Canada; Dalla Lana School of Public Health, University of Toronto, 155 College St, Toronto, Canada; Department of Family and Community Medicine, University of Toronto, 500 University Avenue, Toronto, Canada; Department of Family and Community Medicine, St. Michael’s Hospital, 30 Bond Street, Toronto, Canada; Department of Family and Community Medicine, University of Toronto, 500 University Avenue, Toronto, Canada; Department of Family and Community Medicine, St. Michael’s Hospital, 30 Bond Street, Toronto, Canada; Institute for Health Policy, Management & Evaluation, University of Toronto, 155 College St, Toronto, Ontario, Canada; Department of Family and Community Medicine, University of Toronto, 500 University Avenue, Toronto, Canada; MAP Centre for Urban Health Solutions, Li Ka Shing Knowledge Institute of St. Michael’s Hospital, 30 Bond Street, Toronto, Canada; Department of Family and Community Medicine, St. Michael’s Hospital, 30 Bond Street, Toronto, Canada

**Keywords:** Audit and feedback, Primary care, Practice improvement, Physician learning, Implementation

## Abstract

Explore characteristics of the facilitator, group, and interaction that influence whether a group discussion about data leads to the identification of a clearly specified action plan. Peer-facilitated group discussions among primary care physicians were carried out and recorded. A follow-up focus group was conducted with peer facilitators to explore which aspects of the discussion promoted action planning. Qualitative data was analyzed using an inductive-deductive thematic analysis approach using the conceptual model developed by Cooke et al. Group discussions were coded case-specifically and then analyzed to identify which themes influenced action planning as it relates to performance improvement. Physicians were more likely to interact with practice-level data and explore actions for performance improvement when the group facilitator focused the discussion on action planning. Only one of the three sites (Site C) converged on an action plan following the peer-facilitated group discussion. At Site A, physicians shared skepticism of the data, were defensive about performance, and explained performance as a product of factors beyond their control. Site B identified several potential actions but had trouble focusing on a single indicator or deciding between physician- and group-level actions. None of the groups discussed variation in physician-level performance indicators, or how physician actions might contribute to the reported outcomes. Peer facilitators can support data interpretation and practice change; however their success depends on their personal beliefs about the data and their ability to identify and leverage change cues that arise in conversation. Further research is needed to understand how to create a psychologically safe environment that welcomes open discussion of physician variation.

## Teaser

What physicians believe and their ability to focus the conversation on opportunities instead of limitations is a key element of whether team-based audit and feedback leads to action.

**Implications**

**Practice**: Facilitators need to be able to identify and leverage moments of curiosity that arise in group discussion to help drive data-driven practice change.

**Policy**: Policymakers who want to use audit and feedback to improve patient outcomes and quality metrics should explore scalable models of social interaction to support an action-oriented understanding of the data.

**Research:** Future research should be aimed at identifying interventions to encourage feedback seeking behaviur and designing training programs to shift the attributions individuals make about success and failure.

## Introduction

Medicine is a profession that requires gathering, processing, and acting on numerous sources of data and actioning it to provide the best possible care [1]. Family physicians take a pragmatic approach to learning in this environment—they value practical guidance and instant access to information that relates to specific patients [2]. There is a resulting disconnect between the learning mindset of family physicians who approach care “one person at a time” and performance feedback interventions that provide personalized, aggregate data at the practice level in comparison to a target [3]. This disconnect often leads to unproductive engagement with data, whereby family physicians are motivated to improve but experience challenges understanding how aggregate data provides insights into individualized care. The ensuing struggle to identify actions based on performance feedback is a significant barrier to improving quality at the patient level [4].

This “intention to action gap” underscores the need to pair performance feedback with complementary strategies that support engagement and subsequent practice change. Effective performance feedback needs to consider the credibility of the information and the means of delivery (i.e., via supervisor or colleagues), while providing ample opportunity for social interaction during feedback delivery [5]. Social interaction enhances the uptake of feedback through the development of rapport, trust, and mutual respect, which builds a foundation for meaningful dialogue about performance assessment [6]. Furthermore, it provides an opportunity to build capabilities around interpreting and acting on practice-level data through dialogue and modelling. Mutual respect between parties plays a key role in the uptake of feedback [6, 7], highlighting that *who* supports performance improvement efforts matters.

Cooke et al [8] recently developed a program of audit and group feedback, with group discussions as a central feature of the feedback delivery. These group discussions were held between physician peers, with a physician external to the team acting as a group facilitator [8]. The authors usefully highlight the typical cycle of actions and responses [8]; however, it remains unclear whether and how each of these actions impact the desired outcome of practice change. While the authors note that group reflection led to *“change cues”* that pivoted the discussion towards action planning [8], there was no discussion of the active ingredients of the social interaction that promoted action planning. Hence, it remains to be seen whether aspects of the facilitator, the group, and/or the discussion (i.e., the interaction) influenced action-planning during the group discussions. Distilling which of these features promote action planning will be integral to determining which variables could be modified or avoided to support identifying a resulting action from the data.

The St. Michael’s Hospital Academic Family Health Team (SMHAFHT) in Ontario, Canada adopted a group model of performance feedback facilitation to support the experience of family physicians interpreting and developing actions based on practice-level data. The model utilized trained physician peers to leverage credible relationships and build internal capacity. The objectives of this study were to 1) describe how physicians progressed through the performance feedback cycle in a group session facilitated by their peer, and 2) understand how and why the ability to identify an action varied across sites. Focusing on identifying an action as a target outcome allows for broader insights around intention setting in response to audit and feedback [9] as well as the relative value of social interaction as compared to individual reflection, where identifying an action in response to data has been previously identified as a challenge [4].

## Methods

### Study design

We used a qualitative approach to describe how physicians engaged in a facilitated group-feedback session led by their peers and to explore the reasons for variation across sites. The protocol received ethics approval from the St. Michael’s Hospital Research Ethics Board. All participants provided written informed consent prior to focus groups or observations of group discussions.

### Context and setting

Family Health Teams (FHTs) are publicly funded primary care organizations in Ontario, Canada comprised of interprofessional health care providers (e.g., physicians, nurse practitioners, registered nurses, social workers, etc.) who collaboratively provide care for individuals in their community [10]. The SMHAFHT is a large primary care organization with six clinics at the time of study and approximately 49,000 enrolled patients, served by more than 130 health professionals, including 80 staff physicians. Each physician in the SMHAFHT has a “roster” of patients in their own practice and has access to an interprofessional team to support care for that roster. SMHAFHT serves a diverse patient population ranging from double-income professionals to new immigrants, people living in poverty, people experiencing homelessness and populations who have traditionally faced barriers to care. The team cares for more patients with mental illness, addictions, diabetes, HIV, and other chronic conditions than the average practice [11]. The team has an advanced quality improvement (QI) infrastructure [12] including physician QI leads at each site and active team-based improvement initiatives including ones for cancer screening [13], opioid prescribing [14], smoking cessation, and timely access [15].

For years, the team leadership prepared team and clinic-level reports summarizing quality of care. The only measure consistently reported to individual physicians was Third Next Available Appointment (TNA), a standard measure of timely access to booked appointments. TNA was reported to physicians biweekly by email and also reviewed during their annual meeting with the Physician-in-Chief. In 2016, the SMHAFHT began working with FHT physicians to design an individualized practice report for physicians that summarized demographics and quality of care for their rostered population. The first iteration of the report was provided confidentially to physicians in November 2017 for their individual review. In May 2018, we launched a program piloting three different types of social interactions that were introduced alongside the next iterations of the report: structured self-reflection^4^ (introduced in June 2018), peer coaching (introduced in January 2019), and facilitated group discussions (introduced in November 2019). The current study focuses on physician’s experiences with facilitated group discussion.

The report was initially provided to physicians in PDF and paper format via email and their mailbox. By the time of the facilitated group discussions, physicians received the feedback report in an interactive HTML dashboard that included patient-level data. The dashboard included quality of care indicators using data from the electronic medical record (EMR), a practice patient experience survey, manual chart audit, and administrative sources. Indicators covered a variety of areas including timely access, continuity, patient-centredness, cancer screening, diabetes, immunization, and high-risk prescribing (see Appendix 1 for sample dashboard).

### Intervention

Facilitated group discussions were conducted at each of the six clinics affiliated with the SMHAFHT. Discussions were led by two peer physicians from within the same clinic for all except one site. For sites with two facilitators, one of the facilitators acted as a peer coach in the third iteration of the feedback report and was nominated by their peers; the second facilitator was the designated QI lead for the clinic. For the site with a single facilitator, the nominated facilitator was also the designated QI lead and was comfortable proceeding solo due to the small size of the clinic. All group facilitators attended a two-hour training together, which included a review of evidence on audit and feedback, readings and discussion on the R2C2 model [6] and Alberta Physician Learning Program [16] as well as a review of the indicators in the report, how the indicators were derived, common questions physicians had about the indicators, and examples of relevant SMART goals. The intended focus of the group discussions was to identify clinic-level actions the team could undertake to improve care for the populations they served. The training also walked the facilitators through the agenda for a group discussion (see Box 1) and the slide deck that would be provided to them that contained key points and discussion cues to support the group sessions. Facilitators were encouraged to decide on a topic for the session in advance. To support this, an anonymous paper-based survey was distributed at a medical staff meeting that asked physicians to identify their site and rank the top three topic areas that they would be interested in discussing at the facilitated session. Results for the clinic site were provided to the respective facilitators. Facilitators were responsible for selecting a date and time for the group discussion which were designed to be approximately one-hour in length. Discussions were limited to physicians only as physicians across the FHT indicated they were most comfortable taking the first step of discussing and sharing data with their colleagues and then proceeding to engage in a discussion with the broader interdisciplinary team.

**Box 1. Agenda and ground rules for the facilitated group discussions**

Proposed Agenda

1. What is audit and feedback?
2. Linking QI & continuing professional development
3. Review data
4. Identify barriers and facilitators
5. Discuss a goal for your team/clinic
6. Complete commitment to change form & consider next steps

Ground Rules

- Acknowledge reactions to data
- Acknowledge data limitations
- Safe, learning environment
- Focus on what is within our control

### Participant recruitment

Physicians were introduced to the facilitated group sessions by a research team member (TK) at a staff meeting in Fall 2019 and reminded by a follow-up email. All six clinics held one facilitated group discussion in the study time frame (November 2019-January 2020). Due to limited resources, we excluded sites that involved members of the research team (n=2) as well as one group that had not yet determined a date at the time of data collection. Participants provided verbal consent prior to both the observations and the focus group.

### Data Collection

Three of the six group facilitation sessions were audio-recorded and transcribed verbatim. The study research coordinator (KD) with no relationship to the participants attended the sessions to facilitate this. In addition, we conducted one 90-minute focus group with the facilitators to gather their perspectives on the nature of the facilitated discussion, barriers to interpreting the data, and general reflections on attitudes and engagement (see Appendix 2 for focus group guide). Ten facilitators attended the focus group, with representatives from all six sites, while one facilitator was unable to attend due to a scheduling conflict (this facilitator was not affiliated with one of the three observed study sites). This focus group was conducted by an experienced qualitative scientist (LD) who was not affiliated with the SMHAFHT and had no existing relationship with the participants. The study research coordinator (KD) and three additional members of the research team (TK, KW, NR), each of whom were practicing physicians within SMHAFHT, also attended the focus group to support their engagement in data analysis. At the time of the study, KW was the Department Chief, NR held the title of Quality Improvement Director, and TK was the [QI Program Director]. Further elements of reflexivity can be found in Table 1.

**Table 1.** Research reflexivity

Reflexivity constructs	
Personal Reflexivity	How might your experiences, beliefs, and unique perspectives influenced the research and your interpretations?
Interpersonal ­Reflexivity	What relationships did you have with the physicians across the FHT (or in a specific clinic if more relevant) at the time of the study and how might those have affected the research and the people involved? What power dynamics were at play?
Contextual Reflexivity	How were aspects of context influencing the research and people involved?
Author Responses	
LD	I have prior experience conducting evaluations of clinical performance improvement initiatives which has informed my belief that these initiatives aren’t currently having the intended impact and there is opportunity to improve how they support practice change. My perspective as a non-physician allows me to be curious about what physicians believe, their tacit knowledge, the unconscious habits they develop, and their attitudes in relation to improvement.
	I had no prior interactions or relationships with the broader site or study participants beyond this specific work.
	There are several clinical performance feedback initiatives delivered to primary care physicians in Ontario- all from different sources with differing objectives. I am not familiar with the organizational context beyond what was reported in the interviews.
MDN	I identify as a qualitative researcher with a background in social justice and sociology, therefore I am interested in abstracting individual level data to the level of sociological phenomenon. In this project I found myself interested in the interactions between the participants and being curious about how our findings reflected instances of biopolitics and governmentality.
	I did not have a relationship to the physicians/participants in the study. My involvement was limited to data analysis.
	I was brought onto the research project after the data had been collected to assist with data analysis, interpretation and preparation of the manuscript. Since I was not involved in data collection, at times I questioned the accuracy of my interpretations of the data with members of the research team.
NMI	I come to this research with a strong belief that engaging with data to pursue clinical performance improvement is both necessary and challenging.
	I did not have any personal relationships that played a role.
	The primary care context is one characterized by competing priorities and limited resources.
KD	My research experience and training is centred around the social determinants of health, and therefore I had an understanding that there were multiple factors influencing clinical performance and therefore multiple factors would have to be considered for any actions around improvement.
	I did not have a relationship with physicians across the FHT, but the ones that are researchers, already knew me. Since I was interviewing them and I am not a physician nor did I work directly for the FHT, it may have positively impacted their openness to talk about issues they saw with how the data was being collected and used, as well as things that were going on in their clinic. My social positioning may have also made them less open to talk to me, especially if they thought I wouldn’t be empathetic to the demands of their job as physicians.
	One of the clinics was preparing to move and that may have impacted clinic flow, what people felt was doable, and what was a priority in the immediate future, but this study also presented a lot of “firsts” including the dashboard, coaching and group discussion so it seemed to be welcomed by most.
TU	My training is focused on the social determinants of health and health information technology implementation, areas that emphasize multi-factorial complexity and interrelatedness. This is the lens I brought to data analysis in this study.
	My contribution was limited to data analysis and observing one focus group. I was not known to members of the FHT other than Dr. Tara Kiran, study co-author, and my graduate supervisor. Dr. Kiran did not participate in the focus group I attended, nor did she influence the direction of my analytic work beyond orienting me to the study objectives and methodologies. My graduate supervisor did not participate in the focus group I observed, and all transcripts were deidentified prior to analysis. Their response to one survey was deidentified and aggregated with others before analysis. It is possible that my role observing the focus group as an outsider may have made physicians more or less comfortable sharing their views, but overall, I believe I was sufficiently insulated from participants that any dynamics arising from my role were unlikely to impact the research.
	I was involved in the study for such a limited period (2.5 months for data analysis only) and am therefore unable to recall any potentially influential contextual factors.
NR	As the Quality Improvement Director at St. Michael's Hospital during the time of the study, I was deeply interested in the perspectives of the participants as we were in the process of developing insights on how best our providers approached practice data, it’s integrity and motivation for change. As such, my interpretation of the data was likely focused on the benefits it may have on future opportunities for data audit and feedback at the clinical site.
	I was involved in the production of the data dashboard provided to the physicians at the time of the study and provided insights as to what was included in the dashboard for Physicians to review. Additionally, the Quality Improvement Director is a leader in the department and as such there may be a subjective power dynamic in light of the operational decisions that the Quality Improvement Director may be privy to and the types of practice data physicians have access to.
	The context of the study was in a high-functioning Family Health Team, where interprofessional care is highlighted, as such reflections on data and change ideas may be influenced by the dynamics of local, site-based interprofessional dynamics and access. During the study, one of the six sites had just opened its doors two years prior with a new team of physicians. As such, physicians at this site may have just had their first encounter with practice data and willingness to change may have been influenced by the relatively early level of knowledge of their practices.
KW	I am very open to the process of providing data for reflection and action and my bias towards others should feel similarly. As Department Head, I wanted this project to succeed and for individuals to use data effectively to improve patient care.
	As the Department Head, I endorsed and promoted this research project. I did not directly coach any of my colleagues and I was unable to attend my team’s meeting when the data was discussed. Some of the physicians did bring their practice data voluntarily to review at our annual review meeting.
	I believe our department has a strong culture of quality improvement and commitment to academic medicine. Engagement in quality improvement efforts is voluntary but encouraged. We try to take an appreciative and collaborative approach rather than top down. I hope this is what department members experience however some may feel otherwise.
TK	I am a strong believer in the potential for data driven improvement in primary care. As a health system researcher, I am aware of the gaps in primary care quality, understand the potential for team-based care, and understand how our team performs relative to others (and our relative resources). I helped develop the system for collecting and reporting the measures that the groups reflected on. I have also led many initiatives with our team to improve in related areas and see the possibilities to do things differently. I am a family physician within the same large Family Health Team but do not practice at the clinics featured — however I have knowledge of their culture and processes and history.
	I have been a mentor to several of the focus group facilitators. I was the QI Program Director and several of the QI leads reported to me when I held that role. I have friendly relations with all and have socialized outside of work with a couple. I was present at the group in my clinic and my presence may have influenced how that group was conducted (although I didn’t speak individually to the facilitators in advance). Also, for that reason, we did not include that site in our case study. I was not present at the other clinics group discussions. I was present at the facilitator focus group and my presence may have influenced what was said by them. I have a collaborative leadership style where I aim to build trust and dialogue.
	COVID-19 influenced the potential for iterating to improve, spread, and/or sustain these groups. One of the sites studied is a relatively new site with a higher proportion of junior physicians — this group may have not felt as involved as others in the creation of our QI program and related metrics and may have perceived them as more “top down”.

The focus group was audio recorded and transcribed verbatim. Only one team member (KD) had access to the audio files, which were password protected and stored on a secure server.

### Data analysis

Qualitative focus group data was analyzed using an inductive-deductive thematic analysis approach [17]. Inductive codes were first developed and then deductively categorized according to constructs from the conceptual model of physician responses to audit and group feedback developed by Cooke et al [8]. Two members on the research team (KD and TU) first independently read and re-read the four transcripts (three cases and facilitator focus group) to familiarize themselves with the depth and breadth of the content. After data immersion, codes describing participant’s interactions with the intervention were generated independently. Independently generated codes were then compared and refined with the involvement of a third researcher (LD) to review and help clarify the codebook. Once clarity on the codebook was reached, it was imported into NVivo Version 12 [18] for one team member (KD) to electronically code the remaining transcripts, regularly meeting with another team member (LD) to review and discuss findings.

After all transcripts were coded, the codebook was exported from NVivo and reorganized manually using Microsoft Word into case-specific codebooks by another team member (MDN). We then reviewed the facilitator focus group to extract case-specific data which were coded and added to the case-specific codebooks. Each codebook was organized in line with the conceptual model of physician behaviors in a group feedback session identified by Cooke et al [8], including reactions to the data; understanding and questioning; justifying and contextualizing; reflection and discussion; and change cues. The first step involved creating individual case summaries that included summary statements for each element of the Cooke model [8] as well as summary statements that addressed the outcome of the discussion by asking the question “What was the end result?”. Two team members (MDN and LD) then met to review and finalize the case summaries, which included moving between the transcripts of the group discussions and the facilitator focus group, triangulating data to better understand what happened and its impact on group progress (or lack thereof). The second phase involved comparing the case summaries to identify similarities and differences with the objective of understanding whether and how the elements of the group discussion influenced the outcome. To do this, two members of the research team (MDN and LD) met to compare the cases based on the resulting outcomes (see results). This was facilitated by inserting summary statements onto Miro (online whiteboard platform [19],). We focused specifically on the codes within the *justifying and contextualizing* and *reflection and discussion* themes [8] to explore the study objective of how and why progress differed across the sites. As a final step, we held a virtual analytic meeting with additional members of the research team (TK, NI, KW) to review the findings and invite alternative interpretations. Following the meeting, challenges to interpretations were explored further and the qualitative data was reviewed to ensure accuracy of interpretation and final revision of themes. Qualitative data from the facilitator focus group was iteratively used throughout the analysis process to triangulate and provide further context for the findings from the case-specific summaries.

## Results

A total of 16 participants engaged in one of the three facilitated group discussions, inclusive of the two facilitators at each site (*n* = 6 total). For clarity, we will use the terms facilitator and discussants throughout the results to refer to these two groups. Participant demographics can be found in Table 2.

**Table 2.** Participant demographics

Characteristic	Site A (n=7)	Site B (n=6)	Site C (n=5)
Role	2 facilitators 5 discussants	2 facilitators 4 discussants	2 facilitators 3 discussants
Gender	3 male 4 female	3 male 3 female	0 male 5 female
Career Stage	4 early career 3 mid-career	1 early career 2 mid-career 3 late career	2 early career 1 mid-career 2 late career

*Note: Early career = 0-10 years in practice; mid-career = 11-20 years in practice; late career = 21+ years in practice.*

### Reaction and understanding

Across all cases, the data was met with immediate skepticism. The facilitators decided not to choose a topic in advance, instead proposing that they work with their respective sites to decide on it collaboratively. Discussants often felt like the indicators were not a valid reflection of the care they provide, that there were flaws in the data, or that the wrong metric was being measured, which ultimately contributed to them discounting several indicators (refer to Table 3 for exemplar quotes). While some participants had difficulty interpreting what the data was telling them, most reactions demonstrated the ability of participants to interpret what the data is saying (i.e., their performance in relation to the provided comparators). The majority of discussants pointed to external factors that influenced the data and did not raise how actions of the team may have influenced the outcomes being discussed. Each case demonstrated a unique progression following initial reactions as outlined below.

**Table 3.** Descriptive quotes by case

Case	Theme	Quote
Site A	Justifying and contextualizing	“I mean our urgent [emergency department use] is so much higher than the average and our [patient] income is so much lower and I mean those factors are absolutely going to affect [access], those are reflections of complexity of practice.” D4, Site A
	Deflecting the goal impedes change talk	“The purpose of sharing comparative [third next available appointment (TNA) data] was so that the teams could have conversations about how to support the access of physicians who were having difficulty maintaining low TNA. It was not about shaming which is how the way the vast majority of us have experienced it, I think, and it was not about trying to compare yourself to other people [which] has happened with this data. The data is as good as it is. Our goal shouldn’t be are we as good as everyone else and I think our clinic feels a lot of that ‘we need to be where the rest of the downtown team is’. And certainly the data is being used top down. Why is your clinic not meeting this standard? And I’m not sure that that’s the purpose. Ultimately it should be looking at our data and saying, is this giving us an idea of what we want to improve or do differently?” D9, Site A “If we want team-based care and we want access to team-based care it’s not just access to [physicians], right. If the right thing is to see the social worker or to see the dietician, success would look like that- the patient can’t just get to see me in like seven days or less according to the TNA but there’s another benchmark that we’re meeting like across the team, right. ‘Cause part of improving our access too is making sure that we’re practicing to our appropriate scope of practice along with all of our team members who are able to do the same thing so what can we do, like we’ve done some things where patients can book in directly to see [team members] or they can self-refer for dietician or social worker so that’s great; but if the access for our team members isn’t as good as it is for us then they’re going to come see us instead. So […] how do we know what the access is across the team and then how do we support our interprofessional colleagues in improving their access if it’s not as good as it can be.” D8, Site A
	The outcome	“Yeah, and I mean just to pick up on one of your points, like I think one of the main goals of this session is that we do what you suggested like we, as a group, generate ideas about what’s important to us like what we actually want to focus on and what we want to you know put some effort into actually improving as a group. You know it’s not to like improve all of these or you know adopt all of these and say let’s get better at everything or you know why are we so bad on some things but like to open up that discussion and to, yeah, acknowledge the limits of a lot of these data points but maybe there’s something here that really sticks out to us and I, you know not just the data but our own clinical judgment and our day-to-day.” F2, Site A “Also cause like these are data points that we didn’t necessarily as a group choose to measure; right. These are like selected by someone else and measured for us by someone else so you know part of this maybe is like coming up with what you want to measure for what we care about. Do we care about the number of people who walk in, in like a clinical half-day, do we care about the number of visits like that we do like [Pause] because those things like aren’t being measured and those things in some way probably also relate to access and continuity as well.” F1, Site A
Site B	Justifying and contextualizing	“Yeah, so this is my data actually and I just, I think the reason it kind of climbs steeply over a number of years is I took over another practice and so my numbers went from zero when I started] to about 23 over the first kind of year and a half that I was here and so then I sort of made a choice to scale that back. And so now they’re coming down a bit but I don’t know how much further they can go before I plateau cause some of my folks I just can’t taper.” F1, Site B “60% is nothing to be proud […] but I don’t think there’s a lot that we can do about that. Like the length of time you had to wait is just a function of how busy we are, right.” D4, Site B
	A lack of focus deflected from the goal of the session	“Now if [opioid and benzodiazepine prescribing] is something that we felt interested in taking on as a group we certainly could work with [IT support] to see if we could generate something. We could make sure that he has all of our passwords and see if he’d be able to generate a one-off calculation for us as a group so that we can look at trends over time but my understanding is as it stands now we don’t have team data with respect to these measures.” F2, Site B “I think this has been incredibly helpful because [Pause] I think when we did fill out the first thing as you know we heard that our patients are having difficulty getting in to see us and I mean but when you actually look at the numbers [Pause] not that they’re great but they’re [pretty good].” D4, Site B
	The outcome	“There has been a pharmacist led quality improvement project about ways to approach [opioid prescribing] and she’s actually fantastic. She’s been very helpful to me and will actually counsel patients you know if you raise it with them and they have the tiniest kind of willingness to discuss it she’ll sit down and talk to them about the challenges of being on certain opioids and what we could do instead and so that’s really a great resource […] So that provides support should we wish to pursue that as a challenge.” F1, Site B “Maybe can I just add one more thing because when we had initially said we wanted to focus on access, which was a bit ago, but kind of taking a second look I do agree actually with the smoking piece and I don’t know if everyone wants to relook at this, the smoking stats ‘cause we are not performing I think very well with the smoking piece. I think, I can’t, I don’t know if like when we filled it out we thought it was access and then now taking a second look I also, I’m not in disagreement with what [my colleague] was saying actually around the smoking piece too. There’s a lot of things to be touched on. (D3, Site B) So, I mean I guess one thing we could do cause we really have to wrap up so we’re probably going to have to [Pause] finish this session not completing the task. (F2, Site B)”
Site C	Justifying and contextualizing	“[Patients] are not expecting to, you know, see somebody for quitting smoking- you just throw that into the visit. You know they’re coming in for their arthritis and- I can remember one lady but she’s still heavily smoking- but I’m not going to fill out the [documentation in the] toolbar. It’s an extra step on top of already a very large visit.” D3, Site C “I think that’s okay. I’m just more interested did we ask, I know I’m not asking everybody. And I feel like when I do, you just say yeah, you should quit, here’s some resources like- think about it and that’s it, yeah.” F2, Site C “I think that’s all that matters because I think there’s evidence that us just asking about it actually helps people to think about it and eventually maybe quit-and we can’t make them quit; right. All we can do is-to bring it up.” F1, Site C
	Barriers to reflection slowed progress	“Well it’s more about identifying potential actions and then just deciding what are the barriers and what’s going to help. So, for example if you’re picking smoking cessation then is the barrier the staff- like it’s time, and it’s booking- and so can, a facilitator would be send somebody down to backfill some time to do those things; like to set it as an initiative that’s going to be staffed.” – F2, Site C “So it’s really adding to the clerical load like, every initiative I feel like is going to be a work thing for somebody. So, can we, do we dedicate, do we ask for somebody just to do this for a period of time, like is that a solution, so that you’re not adding workload to the people who are already here [and at capacity].” F2, Site C

### Site A

#### Justifying and contextualizing

The group discussed several indicators but focused largely on those relating to access (including physician-level data on the third next available appointment and group-level data from the practice patient experience survey). While contextualizing the data, participants justified current performance using a range of factors they perceived to be beyond their control, including patient level factors (e.g., the medical and social complexity of our patients impacts access) and process factors (e.g., a lack of a triaging leads to non-urgent concerns being booked into urgent slots).

#### Deflecting the goal impedes change talk

Participants demonstrated curiosity around what they could learn from the data but the discussion centered on the methods behind the report itself rather than what the data was illustrating or describing how their practice was different from others. They expressed uncertainty around the purpose of the data and its comparators and the unintended consequence of shameful emotions that manifested among recipients.

Where moments of more nuanced reflection occurred, the group reflected on the lack of coherence between the perceived team-based (clinic-level) focus of the group session and the individual-level (practice-based) nature of the indicators. This created unresolved tension that was an obstacle to understanding where to go next with participants ultimately articulating the need for more data.

#### The outcome

Participants at Site A struggled to understand the story behind the data, specifically why patients provided the access ratings they did, which influenced their ability to identify an associated action. There was a perceived disconnect between the physician-level data provided on TNA (the indicator the group chose to discuss) and the session’s team-based improvement objective. However, participants overlooked the group-level data in the report including data on patient-reported access. As a result, Site A could not come to a consensus in terms of what indicator to focus on and instead brainstormed how they wanted the practice report to change. This outcome was influenced by a comment by the facilitator who suggested that the indicators were not explicitly chosen by the group and suggested that a new indicator might be an appropriate solution.

Specifically, the group was interested in the addition of new indicators including the number of no shows, same-day cancellations, and patient experience as it relates to phone-based wait times. This was likely also prompted by the facilitators opening the session by asking participants to not only look at the data but also to suggest what should be measured to make the report better. When reflecting on why the session unfolded as it had in the focus group debrief, the group facilitators expressed that the participants at Site A needed to be convinced that the data was useful, and they were unable to achieve this.

### Site B

#### Justifying and contextualizing

Participants discussed several indicators with the general sentiment that the indicators did not reflect the complexity of practice. While contextualizing the data, participants at Site B justified current performance using a range of factors that they perceived to be beyond their control, including patient-level factors and process factors. The high-risk prescribing indicator was seen as primarily reflective of patient factors and therefore beyond the control of both the team and the individual physicians.

When reviewing patient experience survey data, the group justified patient experience of reception room wait times as a function of “how busy they were” and the daily urgent case volume.

#### A lack of focus deflected from the goal of the session

Participants utilized their practice data and experiences attempting practice change as a mechanism to stimulate group reflection. The conversation centered on justifying current performance and feeling that improvement was beyond the participant’s control. The conversation was allowed to evolve organically. The facilitators were able to focus the group on the goal of team-level changes; however, the conversation focused on indicators that lacked team-level data and the group discussed actions to generate these insights.

Interestingly, participants did not discuss variation in indicators among the team members present or discuss physician-level change ideas given this was the nature of available data for the indicators they discussed. Rather, the implicit underlying assumptions were that either patients were receiving high quality care, that care was “pretty good”, or there was nothing within the direct control of the participants that could be done to improve the care they were providing.

#### The outcome

Participants at Site B were caught between the proposed team-based nature of the session and the practice-level nature of the data. Despite successfully identifying a few potential actions over the course of the discussion, including collating different data internally and identifying an existing QI initiative they believed could support individual practice change, the conversation constantly switched focus from one indicator to another and between physician- and group-level actions. As a result, the group did not reach consensus around actions for next steps or a collective team-level change.

### Site C

#### Justifying and contextualizing

Participants discussed several indicators with the general sentiment that the indicators reflected documentation practices and not necessarily the care provided. There was an overarching belief that contextual factors (i.e., small team size, teaching responsibilities) create obstacles to strong indicator-based performance. While the group acknowledged documentation practices (a physician-level factor), it was discussed as a mechanism to justify performance versus an opportunity to improve performance. Interestingly, the group facilitator acknowledged that simply mentioning the topic of smoking cessation might change patient behaviour which would result in improved performance.

Where suboptimal performance was noted, the group felt that the solutions (i.e., after hours and weekend clinics to increase access) had already been implemented but the complexity of the patient population contributed to poor performance.

#### Barriers to reflection slowed progress

There was a perceived need to create a new indicator to reflect the context of individual practices, specifically an indicator that reflected teaching activities that would help participants reflect on the data and confirm their hypotheses driving their justifications of current performance. The facilitator tried to refocus the section by encouraging discussants to reflect on potential actions, modeling what that would look like by reflecting on their own practice.

Group reflection then evolved to acknowledge that engaging in practice changes requires additional work but that everyone is already at capacity. Furthermore, the facilitator reflected that access to care as an indicator introduces feelings of guilt and shame, which creates barriers to ­meaningful group discussion and therefore practice change. This was linked to the notion of being at capacity and the individual feeling that physicians are always staying late to catch up on documentation and administrative paperwork.

#### The outcome

The facilitator was able to validate the skepticism around the data and focus participants at Site C to reach an outcome (i.e., specific action), which involved leveraging an existing clinic resource (a nurse) to address smoking cessation with appropriate patients. It was determined that individual changes were not needed at this juncture given that other clinic-based resources existed to address the problem. This was largely achieved because the facilitator continually refocused the session and did not allow data skepticism to override the conversation. The group identified the need to organize a follow-up meeting with the whole team to reach consensus on the action plan as the proposed change involved engaging a non-physician member of the team who was not present.

### Comparison across cases

Discussions at Sites A and B both involved a considerable focus on justifying and contextualizing the data, including justifying current performance as a product of factors that they perceived to be beyond their control. Both groups also highlighted the tension between the proposed team-based nature of the session and the practice-level nature of the data. The facilitator validated the perspectives of participants but there was an absence of cues to refocus the session on the stated goal.

In contrast, the facilitator at Site C consistently validated the perspectives of participants and followed up these validations with questions and comments to refocus the section on the intended goal of identifying a team-based action. They achieved this by encouraging discussants to reflect on potential actions, modeling what that would look like by reflecting on their own practice. This allowed the group to not only reflect on the data, but also reflect together on the scope and appropriateness of the solution (i.e., have a nurse implement change, revaluate, and see if more individuals need to engage in change efforts). An outcome was identified largely because the facilitator was consistent in their efforts to ­couple ­validating data skepticism with refocus the session and advancing the conversation.

## Discussion

Our findings describe the range of ways groups of physicians progressed through a facilitated feedback discussion led by their peers. Despite similar perceptions of the data across groups, a key inflection point in the discussion occurred when the group either *reflected* on the data and what drives it or *deflected* responsibility for the data. The facilitator’s ability to remain focused on the goal of supporting practice change in one area was central to whether physicians took ownership of the data and explored potential actions for improvement. Among the groups that did not decide on an improvement plan, physicians were persistently skeptical of the data, were either defensive or accepting of performance as good enough, and justified performance as a product of external factors beyond their control. Although one site was able to identify an action in response to the data (engage nurses to address smoking cessation), none of the groups explored variation in indicator performance among physicians attending the group or how changes in physician behavior might influence outcomes.

Comparing our results to Cooke et al [8] raises some key hypotheses around best practices for the co-design of A&F as our results suggest the data included in the reports may not have been perceived as useful or high priority despite early co-design efforts. While clinicians were engaged in the co-creation of reports in both cases, four key elements of the Cooke et al [8] approach likely enhanced the clinical relevance, credibility, and the potential impact of the data: 1) the bottom-up approach to forming the clinical question, whereby a physician or physician group raised a specific clinical question they would like to explore; 2) clarification of the perceived practice gap prior to report development; 3) focusing the report on a single clinical topic; and 4) a facilitator external to the team. While XXXX physician leaders led the development of indicators, it was over a period of several years and not all colleagues involved in the group discussion were similarly engaged in the process. This raises the question of how best to approach the co-design of A&F interventions, including who should be engaged, to align with the way physicians and teams think about the care they provide. For instance, results from our study demonstrate that different discussants had different priorities, creating challenges for reaching a consensus on which indicator to focus on. This suggests that group A&F may be more amenable to facilitation by an experienced external facilitator and focus on a single topic area, ideally identified by the group as operationalized by Cooke et al.[8].

While the focus of the intervention was on team-level changes, our findings also illustrated that physicians in this context are not readily able to identify whether and how their individual or team actions contribute to population-level performance data [4, 9, 20]. Relatedly, it was difficult for them to identify actions to improve performance, as many felt they were doing the best they could as individuals and as a team. The resulting misalignment between the focus of the ­intervention and physicians’ existing skill sets and perceived job role (i.e., focusing on care patient-by-patient) undermines potential impact [21]. This suggests that co-design efforts should extend to include the development of strategies that address this misalignment [22]. Furthermore, the co-design process enabled Cooke et al. to determine and communicate the topic of the session in advance. While the facilitators in this study were encouraged to follow this structure, many elected to have a team-based discussion around which indicator to choose. Future work should explore the impact of both approaches, with consideration for whether the generalist nature of primary care requires a unique approach and the need to train facilitators to effectively navigate scenarios where the preference is to engage the entire group to decide which indicator to focus on. It is also worth noting that when an action was identified (Site C), it was assigned to a member of the team who was not present for the group discussion. This raises a question around the degree of alignment (or misalignment) of the intended outcome (identify a team-level action), the QI strategy (a confidential A&F report delivered to physicians), and the co-intervention (facilitated group feedback with physicians only). Extrapolating best practices from Cooke et al. to the interdisciplinary team context, future initiatives should explore the impact of a process that engages all members of the team in conversations when the intended target is a team-level goal to establish team-level coherence and buy-in.

The characteristics of the facilitators influenced the outcome of the group discussion, underscoring that credibility with the target audience and being a champion of the audit and feedback (A&F) intervention are central to identifying a clear action. A “champion” is an implementation role whereby the individual is: 1) internal to the organization; 2) has an intrinsic interest and commitment to implementing change; 3) works diligently to drive implementation forward; 4) is enthusiastic and persistent; and 5) has strength of conviction [23]. While all facilitators had credibility, those from Sites B and C focused the conversation around how to use the data in the report to inform practice improvements, while the facilitator from Site A endorsed the group suggestion that different data was needed. The progress of Site C further illustrates that what the facilitator *says* or *does* can support reflection and drive the conversation to an actionable outcome. Specifically, the facilitator for Site C provided space to validate physician concerns about the A&F, while also modelling self-reflection in relation to the topic (*“I’m more interested in did we ask, I know I’m not asking everybody”*). This adds to the literature by highlighting the need to train facilitators in how to model reflection and build their skills in linking data to point-of-care actions [8], while simultaneously leveraging peer encouragement and a teaching environment [24]. In parallel, it underscores the need for future work to explore the impact of culture on the effectiveness of an internal or external facilitator. Specifically, whether internal facilitators are limited in their ability to see things objectively and how they balance preserving personal relationships with pursuit of the goal.

Feedback-seeking behavior has important implications for the adaptation of learning and individual performance [25]. Physicians seek information in response to point-of-care questions [26], which creates a disconnect with the population-level focus of A&F and requires a shift in mindset in order to reflect on and identify team-level actions. Perception and interpretation of clinical scenarios are influenced by the tacit knowledge that physicians hold [27], which, by definition, are subconscious. Information-seeking skills are similarly influenced by intrinsic factors, including not recognizing the gap in one’s knowledge and a lack of skills to identify it [28]. It is the measurement of reproducible performance that helps experts develop and maintain their status by identifying aspects of their performance that can be improved [29]. Experts immediately engage in pattern recognition when presented with complex problems, rather than engaging in active thinking, underscoring the need for feedback mechanisms to facilitate monitoring of their performance [29]. While physicians may develop useful shortcuts and heuristics by necessity due to the large volume of information required in medicine, they do not have a consistent mechanism to understand what is out of date or requires improvement. We have argued previously that a paradigm shift is required to support the uptake and impact of A&F at a population level [4]. Reframing A&F as a reflective tool to identify individual- and team-level actions (rather than external factors) that contribute to suboptimal performance is a step towards shifting to an internal locus of control to support behavior modification [30]. Moreover, there is broader need to create a psychologically safe environment, encourage feedback seeking behavior, and design training programs to shift the attributions individuals make about success and failure [25].

The goal of this study was to understand how physicians progressed through the performance feedback cycle in a group session facilitated by their peers. However, there are several limitations to note. While our results highlighted that characteristics of the facilitator were a key driver of how the discussion progressed and whether an action was identified, it was beyond the scope of this study to achieve an in-depth understanding of their beliefs and capabilities. It is interesting to note that one facilitator at each site had been nominated by their peers, suggesting that who physicians prefer to lead conversations may not be the most effective at producing the desired outcome. It is important to note that many of the sites had planned a follow-up group discussion, but these plans were disrupted by the onset of the COVID-19 pandemic, which limited our ability to understand the impact of the original discussions or explore progress over time (as follow-up discussions were postponed due to the system pressures introduced by COVID-19). While we identified that the role of the peer facilitator was a central driver of reflection (or lack thereof), we did not conduct an independent evaluation of the training itself, and are therefore unable to comment on its effectiveness in line with our findings. The next step of this work is to systematically co-design an A&F intervention and complementary co-interventions and evaluate their ability to effectively address the identified barriers (i.e., lack of alignment with what physician’s think is important and capability to draw connections between behavior and practice patterns). Participants in the current study volunteered to participate in a group discussion about the A&F data, therefore the results are not reflective of physicians who opt not to engage with A&F, or prefer a different method of social interaction. It is also important to note that the three excluded sites that we did not observe had identified a goal. Future research should explore what aspects of team culture influence the ability of a group to learn from data and collectively agree on a potential action. Furthermore, while the objective of the group discussions was to identify a team-level action, interdisciplinary team members were not included as part of the discussion which may have limited the breadth of the discussion and undermine buy-in related to implementing the desired action. Given that recruitment was located to a single ­academic center, future work should explore whether and how group-based social interactions are constructed differently in other settings. This work is essential to understanding how A&F and associated co-interventions should be designed and delivered to support quality improvement within and across systems.

## Conclusion

With the accelerating shift to use data to inform practice improvements, there is a pressing need to understand how to create scalable and sustainable models of A&F. While leveraging credible peers is a scalable strategy, the perspectives and skills of peers acting in a facilitator capacity are key drivers of success (or failure). These include leveraging participant’s intrinsic motivation to improve, selecting one topic area of focus at the outset, and a facilitator’s ability to support physicians to look beyond imperfections in the data, redirect conversation, and model the connection between reflection and action. Our findings reinforce how challenging it is for family physicians to identify how physician behavior can be modified to influence practice-level measures.

To better realize the potential impact of A&F, future efforts should shift to designing the necessary co-interventions to help recipients build the skills needed to effectively interpret and act on the data at the physician and team level. Specifically, there is a need to reduce physician defensiveness, explicitly address how imperfect data can still provide insight, and build understanding of how physician behavior influences individual-level care, but also system performance together with other factors. Framing A&F as a helpful mechanism to identify habits and heuristics may also support physicians in shifting their thinking from patient-by-patient care to a population level, which requires a psychologically safe team culture where variation and related drivers can be explored in a supportive and nonjudgmental manner. Facilitators must be credible while also holding the belief that A&F is a good way to achieve QI and modeling reflection that connects the data to individual actions.

## Supplementary Material

ibac114_suppl_Supplementary_Appdendix_A1

ibac114_suppl_Supplementary_Appdendix_A2

## References

[1] Dawes M. Critically appraised topics and evidence-based medicine journals. Singapore Med J. 2005;46(9):442–448; quiz 449. Available at https://www.ncbi.nlm.nih.gov/pubmed/16123827 16123827

[2] MacLeod S. How GPs learn. Educ Prim Care. 2009;20(4):271–277. 19689847

[3] Ivers N , Barnsley J, Upshur R, et al. “My approach to this job is... One person at a time”: perceived discordance between population-level quality targets and patient-centred care. Can Fam Physician Med Fam Can. 2014;60(3):258–266. PMC3952764 24627384

[4] Desveaux L , Ivers N, Devotta K, Ramji N, Weyman K, Kiran T. Unpacking the intention to action gap: a qualitative study understanding how physicians engage with audit and feedback. Implement Sci. 2021; doi:10.1186/s13012-021-01088-1 PMC7891166 33596946

[5] Brehaut JC , Colquhoun HL, Eva KW, et al. Practice feedback interventions: 15 suggestions for optimizing effectiveness. Ann Intern Med. 2016;164(6):435. 26903136 10.7326/M15-2248

[6] Sargeant J , Lockyer J, Mann K, et al. Facilitated reflective performance feedback: developing an evidence-and theory-based model that builds relationship, explores reactions and content, and coaches for performance change (R2C2). Acad Med. 2015;90(12):1698–1706. 26200584 10.1097/ACM.0000000000000809

[7] Telio S , Ajjawi R, Regehr G. The “educational alliance” as a framework for reconceptualizing feedback in medical education. Acad Med. 2015;90(5):609–614. 25406607 10.1097/ACM.0000000000000560

[8] Cooke LJ , Duncan D, Rivera L, Dowling SK, Symonds C, Armson H. How do physicians behave when they participate in audit and feedback activities in a group with their peers? Implement Sci. 2018; doi:10.1186/s13012-018-0796-8 PMC6069557 30064509

[9] Brown B , Gude WT, Blakeman T, et al. Clinical Performance Feedback Intervention Theory (CP-FIT): a new theory for designing, implementing, and evaluating feedback in health care based on a systematic review and meta-synthesis of qualitative research. Implement Sci. 2019; doi:10.1186/s13012-019-0883-5 PMC6486695 31027495

[10] Glazier RH , Redelmeier DA. Building the patient-centered medical home in Ontario. JAMA. 2010; doi:10.1001/jama.2010.753 20516421

[11] Social Determinants of Health. 2021. Available at https://unityhealth.to/social-determinants-of-health/

[12] Kiran T , Ramji N, Derocher M, Girdhari R, Davie S, Lam-Antoniades M. Ten tips for advancing a culture of improvement in primary care. BMJ Qual Saf. 2019;28(7):582–587. 10.1136/bmjqs-2018-008451 PMC6593644 30381328

[13] Feldman J , Davie S, Kiran T. Measuring and improving cervical, breast, and colorectal cancer screening rates in a multi-site urban practice in Toronto, Canada. BMJ Qual Improv Rep. 2017; doi:10.1136/bmjquality.u213991.w5531 PMC5411724 28469908

[14] Tilli T , Hunchuck J, Dewhurst N, Kiran T. Opioid stewardship: implementing a proactive, pharmacist-led intervention for patients coprescribed opioids and benzodiazepines at an urban academic primary care centre. BMJ Open Quality. 2020; doi:10.1136/bmjoq-2019-000635 PMC7170545 32269056

[15] Davie S , Kiran T. Partnering with patients to improve access to primary care. BMJ Open Quality. 2020; doi:10.1136/bmjoq-2019-000777 PMC7170539 32241765

[16] Cooke LJ , Duncan D, Rivera L, Dowling SK, Symonds C, Armson H. The Calgary Audit and Feedback Framework: a practical, evidence-informed approach for the design and implementation of socially constructed learning interventions using audit and group feedback. Implement Sci. 2018; doi:10.1186/s13012-018-0829-3 PMC6208022 30376848

[17] Braun V , Clarke V. Using thematic analysis in psychology. Qual Res Psychol. 2006;3(2):77–101.

[18] NVivo. 2022. Available at https://www.qsrinternational.com/nvivo-qualitative-data-analysis-software/home

[19] Miro. 2022. Available at https://miro.com/

[20] Shea CM , Turner K, Albritton J, Reiter KL. Contextual factors that influence quality improvement implementation in primary care: the role of organizations, teams, and individuals. Health Care Manage Rev. 2018;43(3):261–269. 29533271 10.1097/HMR.0000000000000194 PMC5976517

[21] Patel B , Usherwood T, Harris M, et al. What drives adoption of a computerised, multifaceted quality improvement intervention for cardiovascular disease management in primary healthcare settings? A mixed methods analysis using normalisation process theory. Implement Sci. 2018; doi:10.1186/s13012-018-0830-x PMC6233504 30419934

[22] Hunter, B., Biezen, R., Alexander, K, et al. Future Health Today: codesign of an electronic chronic disease quality improvement tool for use in general practice using a service design approach. BMJ Open. 2020; doi:10.1136/bmjopen-2020-040228 PMC7751202 33371024

[23] Miech EJ , Rattray NA, Flanagan ME, Damschroder L, Schmid AA, Damush TM. Inside help: an integrative review of champions in healthcare-related implementation. SAGE Open Med. 2018; doi:10.1177/2050312118773261 PMC5960847 29796266

[24] Kahouei M , Alaei S, Panahi SSGS, Zadeh JM. Evaluation of organizational readiness in clinical settings for social supporting evidence-based information seeking behavior after introducing IT in a developing country. J Evid-Inf Soc Work. 2015;12(5):500–508. 25839913 10.1080/15433714.2014.921587

[25] Crommelinck M , Anseel F. Understanding and encouraging feedback-seeking behaviour: a literature review. Med Educ. 2013;47(3):232–241. 23398009 10.1111/medu.12075

[26] Cook DA , Sorensen KJ, Wilkinson JM, Berger RA. Barriers and decisions when Answering clinical questions at the point of care: a grounded theory study. JAMA Intern Med. 2013; doi:10.1001/jamainternmed.2013.10103 23979118

[27] Mamede S , Schmidt HG, Rikers R. Diagnostic errors and reflective practice in medicine. J Eval Clin Pract. 2007;13(1):138–145. 17286736 10.1111/j.1365-2753.2006.00638.x

[28] Daei A , Soleymani MR, Ashrafi-Rizi H, Zargham-Boroujeni A, Kelishadi R. Clinical information seeking behavior of physicians: a systematic review. Int J Med Inf. 2020; doi:10.1016/j.ijmedinf.2020.104144 32334400

[29] Ericsson KA. Acquisition and maintenance of medical expertise: a perspective from the expert-performance approach with deliberate practice. Acad Med. 2015;90(11):1471–1486. 26375267 10.1097/ACM.0000000000000939

[30] Payne VL , Hysong SJ. Model depicting aspects of audit and feedback that impact physicians’ acceptance of clinical performance feedback. BMC Health Serv Res. 2016; doi:10.1186/s12913-016-1486-3 PMC4944319 27412170

