# Supplementary material for: Snakes and ladders: A qualitative study understanding the active ingredients of social interaction around the use of audit and feedback
Source: Transl Behav Med. 2023 Jan 24;13(5):316–26. doi: 10.1093/tbm/ibac114 (PMC10182419; doi:10.1093/tbm/ibac114)
Supplement: ibac114_suppl_Supplementary_Appdendix_A2 [file ibac114_suppl_supplementary_appdendix_a2.docx]

**Appendix: Facilitator Focus Group Guide**

1. I’d like to start by asking you to think about the physicians you facilitated in your groups. With that in mind, can you walk me through how the facilitated groups unfolded? What did the group discussion cover and how did it evolve?
   1. How did it support physicians in interacting with and interpreting their data?
   2. What surprised you? What went as planned?
   3. Did anyone share their data with the group? Do you think the discussion impacted people’s openness to sharing data?
   4. Were there any conversations around how to act on the data?
   5. How was the process of coming up with an action plan? How confident are you that your group will be able to follow through?
   6. What, if any, impact did the facilitating have beyond interpreting the data and coming up with an action plan?
   7. Would you do anything differently?
2. What barriers to interpreting and/or acting on the data arose in your group?
   1. Prompt: patient level of ‘one at a time’ vs. population view
   2. Prompt: the nature of primary care (e.g. heterogeneity) vs the attempts of standardization
3. How do you think your colleagues’ view receiving and reflecting on data?
   1. Do you get the sense that people feel it’s a part of their role as a physician to reflect on the care that they provide? The care their patients are receiving? To use data to enhance that reflection?
   2. Do you get the sense that physicians feel it is part of their role to work with others to improve care in their own practice? In the clinic/FHT?
   3. Did facilitating the groups change your own views on the value of data for learning and improvement?
